# *MmpL* Genes Are Associated with Mycolic Acid Metabolism in Mycobacteria and Corynebacteria

**DOI:** 10.1016/j.chembiol.2012.03.006

**Published:** 2012-04-20

**Authors:** Cristian Varela, Doris Rittmann, Albel Singh, Karin Krumbach, Kiranmai Bhatt, Lothar Eggeling, Gurdyal S. Besra, Apoorva Bhatt

**Affiliations:** 1 School of Biosciences, College of Life and Environmental Sciences, University of Birmingham, Edgbaston, Birmingham B15 2TT, United Kingdom; 2 Institute for Biotechnology 1, Research Centre Juelich, D-52425 Juelich, Germany

## Abstract

Mycolic acids are vital components of the cell wall of the tubercle bacillus *Mycobacterium tuberculosis* and are required for viability and virulence. While mycolic acid biosynthesis is studied extensively, components involved in mycolate transport remain unidentified. We investigated the role of large membrane proteins encoded by *mmpL* genes in mycolic acid transport in mycobacteria and the related corynebacteria. *MmpL3* was found to be essential in mycobacteria and conditional depletion of MmpL3 in *Mycobacterium smegmatis* resulted in loss of cell wall mycolylation, and of the cell wall-associated glycolipid, trehalose dimycolate. In parallel, an accumulation of trehalose monomycolate (TMM) was observed, suggesting that mycolic acids were transported as TMM. In contrast to mycobacteria, we found redundancy in the role of two *mmpL* genes, in *Corynebacterium glutamicum*; a complete loss of trehalose-associated and cell wall bound corynomycolates was observed in an *NCgl0228*-*NCgl2769* double mutant, but not in individual single mutants. Our studies highlight the role of *mmpL* genes in mycolic acid metabolism and identify potential new targets for anti-TB drug development.

## Highlights

► *MmpL3* is an essential gene in mycobacteria ► MmpL3 is involved in mycolic acid transport in mycobacteria ► Two *mmpL* genes are involved in corynomycolate metabolism in corynebacteria

## Introduction

Mycolic acids are vital components of the waxy cell wall of *Mycobacterium tuberculosis*, the causative agent of tuberculosis (TB). Synthesized by the concerted and sequential action of three enzymatic units, fatty acid synthase I (FAS-I), fatty acid synthase II (FAS-II), and a polyketide synthase (Pks13), these very long-chain α-alkyl, β-hydroxy fatty acids are essential for viability and virulence (Bhatt et al., 2005, 2007; Brown et al., 2007; Dubnau et al., 2000; Glickman et al., 2000; Vilchèze et al., 2000). While core mycolate biosynthesis is now well studied in mycobacteria and in the related *Corynebacterium glutamicum*, the processing of newly synthesized mycolic acids and their subsequent transport for deposition in the cell wall remains poorly understood. An earlier report of an ABC transporter mutant affected in mycolic acid composition turned out to be an indirect effect due to altered lipoglycan biosynthesis (Mishra et al., 2008; Wang et al., 2006). Takayama et al. (2005) suggested a hypothetical pathway that involves the transfer of a mature mycolate to an isoprenoid carrier, forming MycPL (6-O-mycolyl-β-D-mannopyranosyl-mono phospho- heptaprenol), and subsequently to trehalose inside the cell to yield trehalose monomycolate. TMM is then proposed to be transported by a yet unknown mechanism outside the cell where it is used as a substrate by the mycolyltransferase enzymes of the Ag85 complex (Belisle et al., 1997; Puech et al., 2002), to transfer the mycolate residue to either the arabino-galactan (AG) complex (wall bound mycolates), or to another TMM (resulting in formation of TDM). Alternatives to this hypothetical pathway include intracellular synthesis of TDM prior to transport, or the flipping of Myc-PL to the outside for subsequent use as a substrate for extracellular formation of TMM. Apart from the identification of Myc-PL as a carrier-bound intracellular intermediate (Besra et al., 1994), and the mycolyltransferase activity of the Ag85 proteins (Belisle et al., 1997; Puech et al., 2002), not much is known about the intracellular processing and subsequent transport of mycolic acids.

The genomes of *M. tuberculosis* H37Rv and other mycobacteria contain a number of genes encoding proteins that belong to a family of multidrug resistance pumps termed RND (resistance, nodulation, and division) proteins (Cole et al., 1998; Domenech et al., 2005). Designated MmpL (for mycobacterial membrane protein large), these proteins typically contain 12 transmembrane (TM) domains and two non-TM loops. None of the mycobacterial MmpLs studied so far appear to play any role in drug resistance (*mmpL7* conferred resistance to isoniazid when overexpressed in *Mycobacterium smegmatis* but the *M. tuberculosis mmpL7* mutant did not display altered sensitivity to the drug; Pasca et al., 2005). Instead, many *mmpL* genes are associated with clusters involved in the biosynthesis of cell wall-associated glycolipids like sulfolipids, polyacylated trehalose, glycopeptidolipids, lipooligosaccharides, and other complex lipids like phthiocerol dimycocerosate (PDIM) (Cole et al., 1998; Converse et al., 2003; Cox et al., 1999; Domenech et al., 2004, 2005; Rombouts et al., 2011; Sondén et al., 2005). MmpLs are proposed to function as scaffolds for the biosynthetic machinery, allowing localized synthesis of a cell wall associated lipid, pairing with an ABC transporter (Sondén et al., 2005), and in some cases an MmpS protein (Deshayes et al., 2010), to facilitate transport. Indeed, a two hybrid screen using the non-TM domains of the PDIM transporter MmpL7 revealed interactions with enzymes involved in the final stages of biosynthesis of the phthiocerol moiety (Jain and Cox, 2005). In some cases, such “mega complexes” may involve localized “flipping” of a biosynthetic intermediate for presentation to extracytoplasmic enzymes involved in the biosynthesis of the lipid, as was observed with MmpL8 and sulfolipid SL-I (Converse et al., 2003; Domenech et al., 2004). Thus, it seemed likely that an MmpL protein may be involved in the translocation of a mycolate-containing glycolipid (TMM, TDM, or Myc-PL) to the outside of the bacterial cell for subsequent use as a substrate for cell wall mycolylation.

*Mycobacterium leprae*, which contains a high degree of “genetic decay” but still possesses an intact cell wall containing mycolic acids, represents a valuable reference when considering candidate genes for essential cell wall-related functions like mycolic acid metabolism. Homologs of only 5 of 14 *M. tuberculosis mmpL* genes are present in *M. leprae*, of which only *mmpL3* is predicted to be an essential gene based on studies in *M. tuberculosis* (Domenech et al., 2005). Given the essentiality of mycolic acids for viability of mycobacteria (Bhatt et al., 2005; Parish et al., 2007; Portevin et al., 2004; Vilchèze et al., 2000), it was likely that *mmpL3* was involved in mycolate transport in *M. tuberculosis*. In this study, we have investigated the role of *mmpL3* in mycolic acid transport by generating and characterizing a conditional mutant of *MSMEG0250*, the homolog of *mmpL3* in the fast growing *Mycobacterium smegmatis*. In parallel, we also compared and contrasted the role of *mmpL* genes in *Corynebacterium glutamicum*, a bacterium that has a cell wall similar to mycobacteria including the presence of mycolic acids, called corynomycolic acids (both belong to the suborder Corynebacterineae). A particular advantage of using corynebacteria is the ability to generate viable corynomycolate-deficient mutants (Gande et al., 2004, 2007; Portevin et al., 2004, 2005) and our studies demonstrated a functional redundancy of corynomycolate metabolism-associated MmpLs in this genus.

## Results

### MmpL3 Is an Essential Gene in Mycobacteria

**Figure 1:**
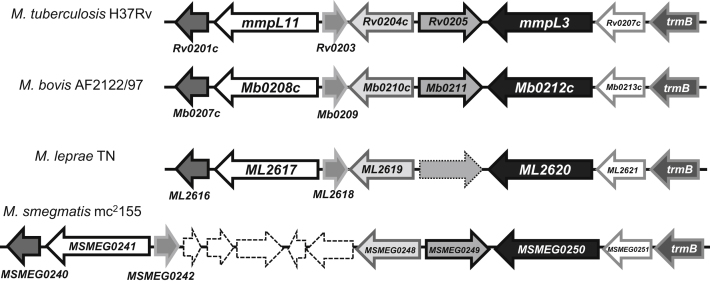
Maps of the *mmpL3-mmpL11* Region in Different Mycobacteria Homologous genes are indicated by similar arrows. Arrows with hatched borders indicate genes found exclusively in *M. smegmatis*, and a pseudogene in *M. leprae* is depicted by an arrow with a dotted border.

*M. tuberculosis mmpL3* (*Rv0206c*) was first proposed to be an essential gene by Domenech et al. (2005) on the basis of the inability to obtain an *mmpL3* knockout mutant. While the gene is not located near any known mycolic acid biosynthesis genes, the gene cluster surrounding *mmpL3* shows a high level of synteny with other mycobacterial species including *M. leprae* (Figure 1). This includes the presence of three putative membrane associated proteins (Figure 1), and a second *mmpL* gene, *mmpL11*, situated downstream of *mmpL3*. While Domenech et al. (2005) did not detect any alterations in the lipid profile of an *M. tuberculosis mmpL11* mutant, the strain was attenuated in the mouse model of infection.

**Figure 2:**
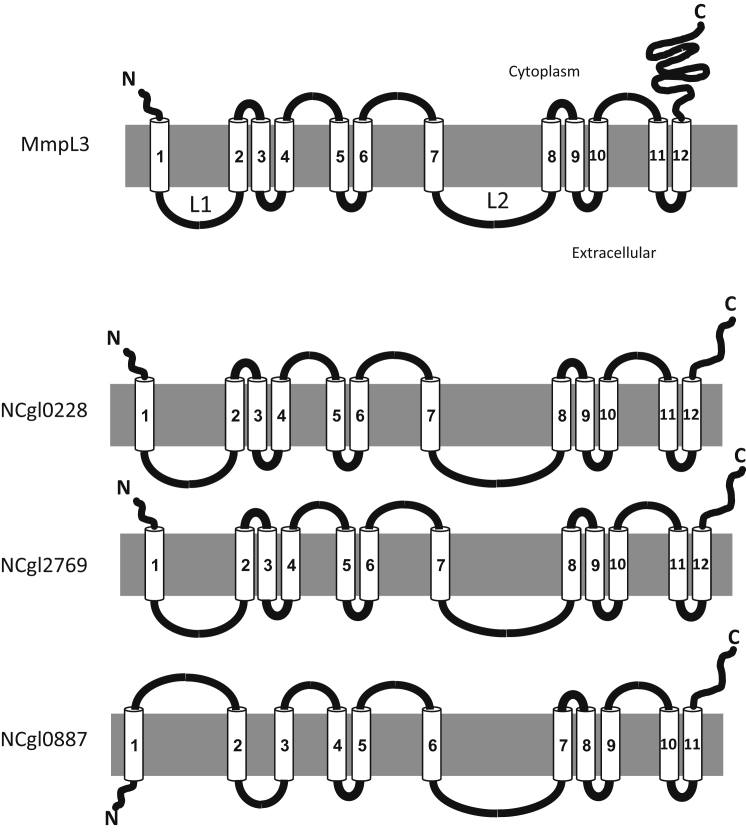
Predicted Topology of MmpL3 and Corynebacterial MmpLs The TM regions are indicated by cylinders and are numbered while the two non-TM loops of MmpL3 are indicated as L1 and L2. The carboxy and amino termini are indicated by “C” and “N,” respectively. The Mobyle@Pasteur server was used for topology predictions (http://mobyle.pasteur.fr/cgi-bin/portal.py#forms::toppred).

**Figure 3:**
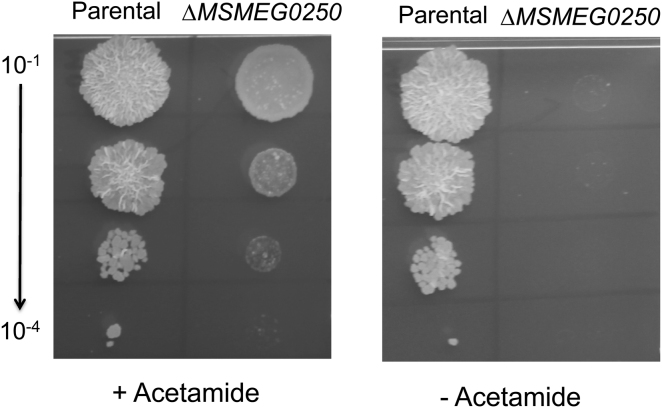
Essentiality of *MSMEG0250* in *M. smegmatis* mc^2^155 Growth of the conditional mutant Δ*MSMEG0250* and parental merodiploid strain (mc^2^155::pMV306*mmpL3*) on Tryptic Soy Agar with or without the inducer acetamide. Ten microliters of 10-fold serial dilutions of cultures was spotted on the agar plates and incubated for 5 days at 37°C.

The protein encoded by *mmpL3* is predicted to be a membrane associated protein, and contains 12 TM domains and two non-TM loops, L1 and L2 of sizes 151 and 142 amino acids, respectively (Figure 2). We first constructed a knockout phage designed to delete *MSMEG0250*, the *M. smegmatis* homolog of *M. tuberculosis mmpL3*, and consistent with findings from previous studies in *M. tuberculosis* (Domenech et al., 2005), we were unable to generate a null mutant. Subsequently, CESTET, a tool designed to test gene essentiality in *M. smegmatis* (Bhatt et al., 2005), was used to obtain a conditional mutant of *MSMEG0250*. The strain, Δ*MSMEG0250* contained a recombinant, integrated copy of *mmpL3* under the control of the inducible acetamidase promoter, while the native copy of the gene was deleted and replaced with a hygromycin resistance cassette. The viability of Δ*MSMEG0250* was dependent on the addition of the inducer acetamide in the growth medium confirming that *MSMEG0250* was an essential gene (Figure 3).

### MmpL3 Is Involved in Mycolic Acid Transport in Mycobacteria

**Figure 4:**
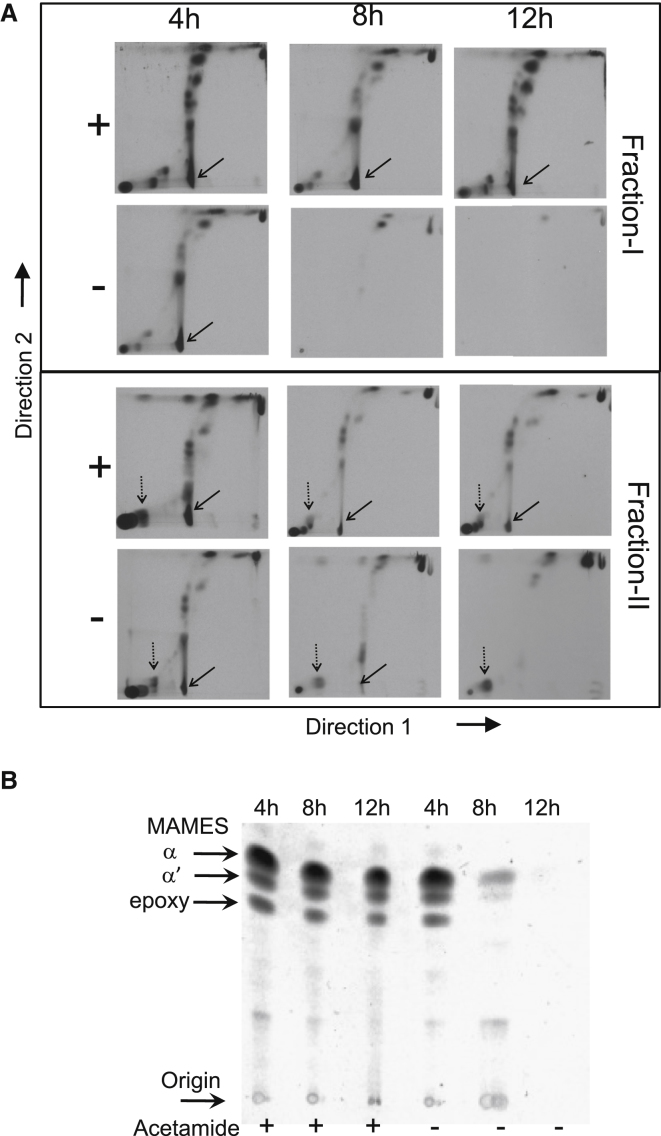
Lipid Analysis of the *ΔMSMEG0250* Conditional Mutant (A) 2D-TLC analysis of [^14^C]-labeled lipids from the *ΔMSMEG0250* conditional mutant. Cultures were grown and labeled in tryptic soy broth in the presence or absence of acetamide. Petroleum ether extracts (Fraction-I) and intracellular apolar lipids (Fraction-II) were separated on using chloroform:methanol:water (100:14:0.8) in Direction 1 and chloroform:acetone:methanol:water (50:60:2.5:3) in Direction 2. Positions of TMM and TDM are indicated by dotted and solid arrows, respectively. (B) TLC analysis of methyl esters of [^14^C]-labeled, cell wall bound mycolic acids (MAMES) separated using petroleum ether:acetone (95:5) as the solvent system. Methyl esters of the different subclasses of mycolic acids are indicated. See also Figure S1.

Recently, spontaneous mutants resistant to BM212 and SQ109, compounds with anti-TB activities, were shown to contain mutations mapping to *mmpL3* (LaRosa et al., 2012; Tahlan et al., 2012). Furthermore, cells exposed to SQ109 were shown to accumulate TMM suggesting that MmpL3 was involved in the efflux of TMM. To address the putative role of MmpL3 in TMM efflux, we assessed the effects of depletion of *MSMEG0250* on lipid metabolism in the *M. smegmatis* conditional mutant, making use of a three stage extraction procedure: (1) petroleum ether extraction of the cell pellet that does not affect the integrity of the cells, but extracts the surface-exposed, noncovalently bound cell wall lipids; (2) extraction of apolar and polar lipids from the “petroleum ether stripped” pellet; and (3) extraction of AG-bound mycolic acids from the resultant delipidated cells. Cultures of the Δ*MSMEG0250* conditional mutant were grown in broth in the presence or absence of acetamide, labeled with [^14^C]-acetate at different time points and subjected to the above three stage extraction procedure. For cultures grown in the presence of acetamide, TMM was predominantly found in the apolar lipid extract from cell pellets (Figure 4A). However, TDM was found in both the petroleum ether extract, and in the apolar lipid extract from the subsequent cell pellets. An earlier examination we had conducted of *M. smegmatis* cell pellets extracted with consecutive treatments of petroleum ether indicated that no further TDM was extracted from cells after the third consecutive extraction. Each petroleum ether extract of the Δ*MSMEG0250* conditional mutant shown in Figure 4A represent a pool of five consecutive extractions of the same sample and thus the presence of TDM in the subsequently processed cell pellet was not due to insufficient petroleum ether extraction. The exact location of these cell-associated TDM molecules that resist petroleum ether extraction is unknown, but it is possible that they represent newly synthesized TDM that is closer to the inner membrane and thus inaccessible to petroleum ether extraction.

In contrast to the cultures of the conditional mutant grown in the presence of acetamide, [^14^C]-labeled cultures grown in the absence of acetamide showed an increasing amount of TMM in cell pellets, that was accompanied by decrease in TDM levels both in the petroleum ether extracts and in the apolar fraction from cells (Figure 4A; Figure S1 available online). Conditional depletion of *MSMEG0250* also resulted in a decrease in mycolylation of AG (Figure 4B). The accumulation of TMM in the cells over time following conditional depletion suggested that loss of *MSMEG0250* function affected TMM transport either directly or indirectly.

### Redundant *mmpL* Functions in *Corynebacterium glutamicum*

The cell walls of corynebacteria, which are similar to those of mycobacteria, contain trehalose monocorynomycolate (TMCM) and trehalose dicorynomycolate (TDCM), the equivalents of TMM and TDM, as the outer, noncovalently bounds glycolipids. As corynomycolic acid biosynthesis is a nonessential process, it is possible to generate viable mutants of corynebacteria that do not produce corynomycolates. In a parallel study on the role of MmpLs in corynomycolate transport in *C. glutamicum*, we conducted a BLAST search of the *C. glutamicum* ATCC 13032 genome, using the amino acid sequences of open reading frames from *M. tuberculosis mmpL3-mmpL11* region as queries. The search revealed that there was no corynebacterial equivalent of the mycobacterial *mmpL3-mmpL11* cluster. However, the genome of *C. glutamicum* does encode four *mmpL* genes, *NCgl0228*, *NCgl0559*, *NCgl0887*, and *NCgl2769* in distinct locations on the genome. As TMCM and TDCM are the only abundant, noncovalently bound outer envelope glycolipids in *C. glutamicum*, it seemed likely that one or more of these *mmpL* genes were involved in corynomycolate transport. As *NCgl0599* was associated with a cluster involved in the biosynthesis of a carotenoid pigment (Krubasik et al., 2001), it was not considered for further study.

**Figure 5:**
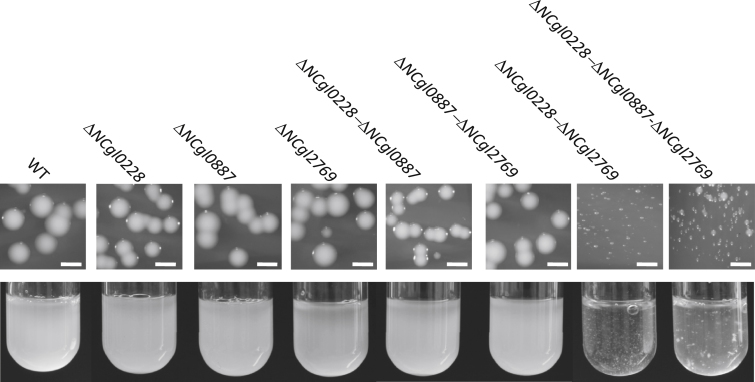
Growth Characteristics of the *C. glutamicum mmpL* Mutant Strains Colonies of *C. glutamicum mmpL* mutant strains on brain heart infusion agar plates (top panel) and broth cultures of the same in brain heart infusion broth (bottom panel). The scale bar represents 1 mm.

NCgl0228 and NCgl2769 revealed predicted topologies similar to *M. tuberculosis* MmpL3 (12 TM domains and two non-TM loops), while that of NCgl0887 appeared different (Figure 2). Interestingly, *NCgl0887* is situated in a cluster containing a trehalose corynomycolyl transferase, and *NCgl2769* is present downstream of the corynomycolate condensase gene, *pks13*. To probe the role of *C. glutamicum mmpLs* in corynomycolate transport, we generated individual in frame deletion mutants of *NCgl0228*, *NCgl0887*, and *NCgl2769* in *C. glutamicum* ATCC 13032. Loss of corynomycolic acids from the cell envelope results in poor growth rates leading to smaller colonies (Gande et al., 2004). None of the three mutant strains (Δ*NCgl0228*, Δ*NCgl0887*, and Δ*NCgl2769*) showed any alterations in colony size or appearance (Figure 5). To investigate potential functional redundancies between the three *C. glutamicum mmpL genes*, double and triple mutant strains were constructed. While the colony sizes and appearance of the double mutants, Δ*NCgl0228*-Δ*NCgl0887* and Δ*NCgl0887*-Δ*NCgl2769*, remained unaltered (Figure 5), the Δ*NCgl0228*-Δ*NCgl2769* mutant formed smaller colonies and showed “clumpy” growth in broth. A similar growth defect was observed for the triple mutant Δ*NCgl0228*-Δ*NCgl0887*-Δ*NCgl2769* (Figure 5). Thus, while single deletions of *NCgl0228* or *NCgl2769* did not have any effect, the concurrent loss of *NCgl0228* and *NCgl2769* severely affected the growth patterns of the mutant strain, indicating potential alterations in the cell wall.

### *NCgl0228* and *NCgl2769* Play a Role in Corynomycolate Metabolism

**Figure 6:**
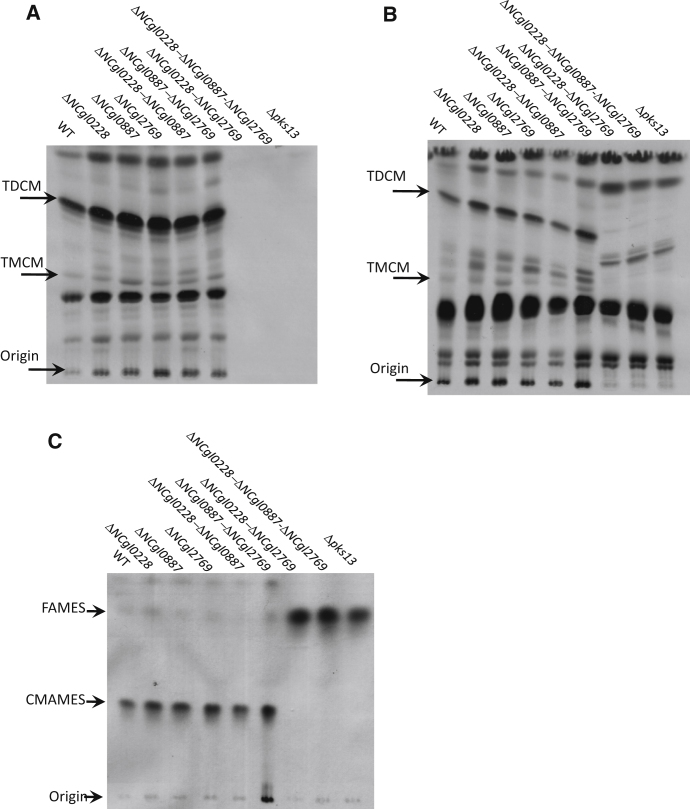
Lipid Analysis of the *C. glutamicum mmpL* Mutant Strains (A and B) TLC analysis of [^14^C]-labeled lipids extracted from *C. glutamicum mmpL* mutant strains separated using CHCl_3_:CH_3_OH:H_2_O (60:16:2) as the solvent system. (A) Petroleum ether extracts, (B) total lipids from petroleum ether-treated cells. (C) TLC analysis [^14^C]-labeled fatty acid methyl esters (FAMES) and corynomycolic acid methyl esters (CMAMES) extracted from delipidated cells, using petroleum ether:acetone (95:5) as the solvent system. The *pks13* null mutant Δ*pks13*, which does not produce corynomycolic acids, was used as a control.

**Figure 7:**
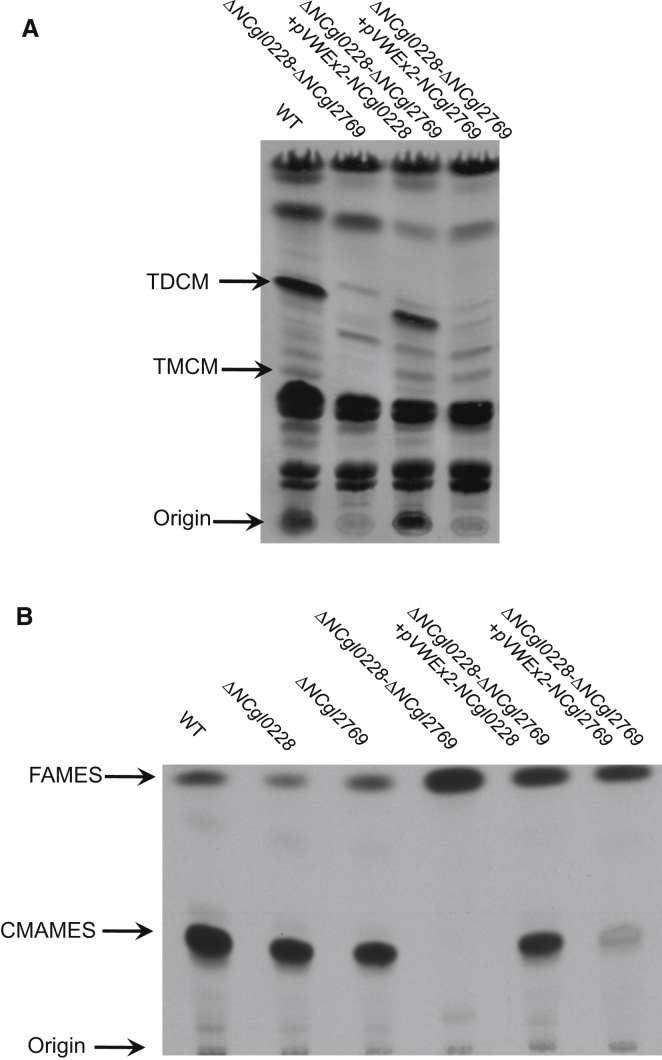
Complementation of the *C. glutamicum mmpL* Double-Mutant Strain TLC analysis of (A) total lipids and (B) AG-bound corynomycolates from complemented *C. glutamicum* Δ*NCgl0228-ΔNCgl2769* double-mutant strains. See also Figure S2.

To determine whether the phenotype observed above for some of the *C. glutamicum mmpL* mutants were linked to defects in corynomycolate transport, petroleum ether extracts of surface-exposed lipids, and subsequent, remaining total lipids were extracted from [^14^C]-acetate-labeled cell pellets. TLC analysis showed that the parental strain and most mutant strains produced TMCM and TDCM, with the latter found predominantly in the petroleum ether extracts containing surface-exposed lipids (Figures 6A and 6B). Furthermore, levels of AG-bound corynomycolic acids for most mutant strains were the same as the parental, wild-type strain *C. glutamicum* (Figure 6C). The two exceptions were the Δ*NCgl0228*-Δ*NCgl2769* and Δ*NCgl0228*-Δ*NCgl0887*-Δ*NCgl2769* mutant strains, in which no TMCM or TDCM was detected in the petroleum ether extracts or in the cell pellets (Figures 6A and 6B). Additionally, no AG-bound corynomycolic acids were observed in the Δ*NCgl0228*-Δ*NCgl2769* and Δ*NCgl0228*-Δ*NCgl0887*-Δ*NCgl2769* strains (Figure 6C). The absence of any TMCM, TDCM, and AG-bound corynomycolates suggested either that there was a complete cessation of corynomycolic acid biosynthesis in the double and triple mutants or that corynomycolates were being transported in an alternate (carrier-bound) form that was used as substrate for TMCM, TDCM, and AG-bound corynomycolate synthesis. However, the latter seemed less likely as no intermediates were seen to accumulate in the Δ*NCgl0228*-Δ*NCgl2769* and Δ*NCgl0228*-Δ*NCgl0887*-Δ*NCgl2769* mutants. These results also indicated a functional redundancy between *NCgl0228* and *NCgl2769* with regards to corynomycolate metabolism. No additional alterations in lipid profiles were observed in the triple mutant suggesting that *NCgl0887* did not play any apparent role (Figures 6A–6C). Complementation of the double mutant with either plasmid-borne *NCgl0228* or *NCgl2769* restored corynomycolate biosynthesis, though in case of the latter gene, the complementation was partial (Figures 7A and 7B and Figure S2).

## Discussion

Our studies demonstrated that *mmpL3* plays a role in mycolic acid transport; the intracellular accumulation of TMM in the *M. smegmatis MSMEG0250* mutant following conditional depletion suggested that TMM was the likely carrier for the mycolic acid moiety. The exported TMM is presumably used as substrate by the enzymes of the Ag85-complex for mycolylation of the cell wall AG and the formation of cord factor (TDM). Our findings were complemented by the drug-to-target approach used by La Rosa et al. (2012) and Tahlan et al. (2012), with the latter demonstrating that treatment with a compound that targeted MmpL3 resulted in a phenotype identical to what we observed with the *M. smegmatis* conditional mutant. Additionally, during the review of this manuscript, Grzegorzewicz et al. (2012) reported that AU1235, an adamantly urea inhibitor of *M. tuberculosis*, also targeted MmpL3. Notably, the authors showed a decrease in the levels of TDM and AG-bound mycolates in *M. tuberculosis* cells treated with AU1235, and accumulation of TMM in the inner membrane. Additionally, they were also able to reproduce the effects of AU1235 in cultures of a *M. smegmatis* conditional *mmpL3* mutant grown under nonpermissive conditions; a result similar to that obtained for the Δ*MSMEG0250* mutant described in this study.

MmpL3 may not be solely responsible for transport; in *M. smegmatis*; glycopeptidolipid transport requires the concerted action of an MmpL protein, MmpS4 and a third transmembrane protein termed Gap (Deshayes et al., 2010; Sondén et al., 2005). The region downstream of *mmpL3* encodes two transmembrane proteins, Rv0204c and Rv0205, which may form part of a complex involved in the transport of TMM (though the *M. leprae* homolog of the latter is annotated as a possible pseudogene). Additionally, the biosynthetic clusters for PDIM also contain ABC transporters and thus the role of ABC transporters in mycolate transport (acting in conjunction with MmpL3) remains a possibility.

A recent study by Tullius et al. (2011) has suggested a role for the *mmpL3-mmpL11* gene cluster in heme transport. The authors report that they were also able to delete a region of the *M. smegmatis* genome that included *MSMEG0250*. In our studies, we were unable to generate an individual null mutant of *MSMEG0250* in *M. smegmatis* mc^2^155 despite repeated attempts (also observed for the *M. tuberculosis* homolog by Domenech et al., 2005). We were, however, able to demonstrate the essentiality of *MSMEG0250* using CESTET and show that conditional depletion caused a loss of TDM and of cell wall (AG) mycolylation, and the intracellular accumulation of TMM.

In contrast to mycobacteria, we observed several differences in the role of *mmpL* genes in corynomycolate transport in *C. glutamicum*. First, there was no equivalent corynebacterial cluster exhibiting synteny with mycobacterial *mmpL3-mmpL11* region. Instead, a functional redundancy was observed between *NCgl0228* and *NCgl2769* located in two distinct regions of the *C. glutamicum* genome. Individual deletion of either of these *mmpL* genes did not have any effect, but simultaneous deletion of both genes led to complete loss of TMCM, TDCM and AG-bound corynomycolates. This was in contrast to the *M. smegmatis* conditional mutant in which conditional depletion of *MSMEG0250* led to loss of TDM and AG mycolylation, but intracellular accumulation of TMM. As mentioned above, while this result raised the possibility that corynomycolate transport may involve a carrier other than TMCM, this seemed highly unlikely, given the absence of any accumulating intermediate in the *C. glutamicum* Δ*NCgl0228*-Δ*NCgl2769* and Δ*NCgl0228*-Δ*NCgl0887*-Δ*NCgl2769* mutant strains. Instead, variations in the phenotypes of the mycobacterial and corynebacterial mutants may be a reflection of fundamental differences in mycolic acid biosynthesis in the two genera. Mycobacterial mycolic acid biosynthesis requires several components including a multidomain type-I fatty acid synthase (FAS), a multienzyme type-II FAS complex, modifying enzymes and Pks13 (Radmacher et al., 2005; Takayama et al., 2005). Corynomycolate biosynthesis on the other hand requires two type-I FASs and Pks13. MmpLs are also envisaged to act as scaffolds for the localized biosynthesis on the cell membrane, facilitating concerted biosynthesis and transport. In mycobacteria, due to the far larger number of components involved, mycolate biosynthesis may be delinked from transport. Instead, MmpL3 could form a complex with other proteins involved in TMM transport. As a result, loss of MmpL3 would not cause a direct cessation of mycolate biosynthesis. In contrast, in *C. glutamicum*, where corynomycolate biosynthesis requires fewer enzymatic components, late-stage enzymes such as Pks13 may depend on interaction with an MmpL-complex to ensure that corynomycolate biosynthesis is coupled to transport. Thus, a concurrent loss of *NCgl0228* and *NCgl2769* would lead to not just the loss of a transport complex, but also disrupt the late stages of corynomycolate biosynthesis. The identification of *mmpL3* as an essential membrane protein-encoding gene involved in mycolate transport opens up new avenues for targeting this vital and under-exploited mycobacterial pathway for developing new anti-TB drugs, an opportunity highlighted by the identification of MmpL3 as the target of three drugs (La Rosa et al., 2012; Tahlan et al., 2012; Grzegorzewicz et al., 2012).

## Significance

**One of the greatest challenges for combating TB is the recent rise of multidrug-resistant (MDR) and extensively drug-resistant (XDR) strains of *M. tuberculosis*. Despite being critical for viability and virulence (and the target of the hallmark anti-TB drug isoniazid), pathways involved in the biosynthesis of mycolic acids remain poorly exploited as drug targets. Additionally, components involved in the postbiosynthesis processing and transport of mycolates are not known and remain an untapped source of potential drug targets. This study identifies a gene (*mmpL3*) involved in mycolic acid transport in mycobacteria. Furthermore, the results obtained from the *C. glutamicum* mutants indicate that transport processes may be coordinated with biosynthesis via protein-protein interactions with MmpLs acting as molecular scaffolds, thus opening up new avenues for studying novel transport mechanisms for mycobacterial lipids.**

## Experimental Procedures

### Generation and Characterization of a *M. smegmatis MSMEG0250* Conditional Mutant

The *M. smegmatis* conditional mutant Δ*MSMEG0250* was generated using CESTET (Bhatt et al., 2005). Briefly, a merodiploid was first generated by introducing an integrating vector pMV306-*mmpL3* by electroporation into *M. smegmatis* mc^2^155 (Snapper et al., 1990) (pMV306-*mmpl3* consists of *mmpL3* cloned downstream of the acetamidase promoter in the integrative vector pMV306 (Stover et al., 1991)). The merodiploid strain mc^2^155::pMV306*mmpl3* was then subjected to specialized transduction as previously described (Bardarov et al., 2002; Larsen et al., 2007) using phΔ*MSMEG0250*, a temperature sensitive, recombinant phage designed to replace *MSMEG0250* with a hygromycin resistance marker. Transductants were selected at the nonpermissive temperature of 37°C on selective plates containing 150 μg/ml hygromycin. After confirmation of gene replacement by Southern blot, one such transductant was named Δ*MSMEG0250* and was selected for further analysis. Conditional depletion of MSMEG0250 in Δ*MSMEG0250* to visualize [^14^C]-labeled mycolic acids and other lipids was carried out as described before (Bhatt et al., 2005).

### Generation and Characterization of *C. glutamicum mmpL* Mutant Strains

To generate specific deletions of *NCgl0228*, *NCgl0887*, or *NCgl2769* in the chromosome of *C. glutamicum*, the nonreplicable vector pK19mobsacB was used (Schäfer et al., 1994), containing inserts synthesized by GeneArt. The inserts contained 12 nucleotides (nt) of the 3′ end of the respective gene together with 300 bp genomic upstream sequences, and 36 nt of the 5′ end together with 300 bp genomic downstream sequences. The respective vector constructed was used to transform *C. glutamicum* ATCC 13032 to kanamycin-resistance (Kan^r^) indicating chromosomal integration. Sucrose-resistant (Suc^r^) clones were selected in a second round of positive selection, indicating loss of the vector-encoded *sacB* function. After the second recombination event about half of the recombinants contained the desired deletion, as verified by PCR, and one strain each was termed *C. glutamicum* Δ*NCgl0228*, *C. glutamicum* Δ*NCgl0887*, and *C. glutamicum* Δ*NCgl2769*, respectively. The double-mutant *C. glutamicum* Δ*NCgl0887*Δ*NCgl2769* was generated using *C. glutamicum* Δ*NCgl0887* and using the appropriate pK19mobsacB construct. In-frame deletion of *NCgl0228* in a single- or double-mutant background was not possible despite repeated attempts. As an alternative, we used pK19mobsacB*NCgl0228*, containing an internal fragment of *NCgl0228* amplified by primer pairs 5′-CATAGAATTCGTGGCTGTGCTCATTGCGTTGAC-3′ and 5′-GTACGTCGACCTCTGCCATCAAATCAGCCGACTG-3′. This vector was used to disrupt *NCgl0228* in *C. glutamicum* Δ*NCgl0887*, in *C. glutamicum* Δ*NCgl2769* and in *C. glutamicum* Δ*NCgl0887-*Δ*NCgl2769* to generate the double-mutants *C. glutamicum* Δ*NCgl0228-*Δ*NCgl0887* and Δ*NCgl0228-*Δ*NCgl2769*, and the triple mutant *C. glutamicum* Δ*NCgl0228-*Δ*NCgl0887-*Δ*NCgl2769*. Growth of all mutants was characterized on the complex medium brain heart infusion broth at 30°C, with 25 μg ml^-1^ kanamycin when appropriate. Extraction of [^14^C]-labeled of lipids and corynomycolic acids from all strains was done as described previously (Gande et al., 2004, 2007).

### Complementation of *C. glutamicum ΔNCgl0228-ΔNCgl2769*

*NCgl0228* was amplified using primer pairs F0228 (5′-CTCATTTGTCGACAAGGAGATATAGGTGGCGAAATTGCTATTCAGG-3′) and R0228 (5′-GGTGGGATCCCTAACGTGCAGCCTGCTTCTCC-3′); and *NCgl2769* using the primer pair F2769 (5′-ACAATTGTCGACAAGGAGATATAGGTGTTTTCTAAATGGGGCCAC-3′) and R2769 (5′-TCCTCGCGGATCCTTAATCTAGATCCTCAAGCCTGC-3′), using *C. glutamicum* ATCC 13032 chromosomal DNA as template. PCR products were cloned in pVWEx2 (Eggeling and Bott, 2005) to yield pVWEx2-*NCgl0228* and pVWEx2-*NCgl2769*, respectively. The inserts in the plasmids were verified by sequencing, and subsequently used to transform *C. glutamicum* Δ*NCgl0228*Δ*NCgl0887* to tetracycline resistance (5 μg ml^-1^) using transformation protocols described by Eggeling and Bott (2005).
