# Supplementary material for: MmpL Genes Are Associated with Mycolic Acid Metabolism in Mycobacteria and Corynebacteria
Source: Chem Biol. 2012 Apr 20;19(4):498–506. doi: 10.1016/j.chembiol.2012.03.006 (PMC3370651; doi:10.1016/j.chembiol.2012.03.006)
Supplement: Document S1. Figures S1 and S2 [file mmc1.pdf]

Chemistry & Biology, Volume *19*

Supplemental Information

MmpL Genes Are Associated with Mycolic Acid Metabolism in Mycobacteria and Corynebacteria

Cristian Varela, Doris Rittmann, Albel Singh, Karin Krumbach, Kiranmai Bhatt, Lothar Eggeling, Gurdyal S. Besra, and Apoorva Bhatt

Inventory of Supplemental Information

Figure S1: This is a graph showing the densitometric estimation of TMM and TDM in the TLCs shown in Figure 4A.

Figure S2: Autoradiograph of a TLC related to Figure 7A. As complementation of one of the strains was partial, fractionation was carried out to concentrate the apolar lipids to better visualise the partial restoration of TMCM and TDCM biosynthesis in the complemented strain.

Figure S1

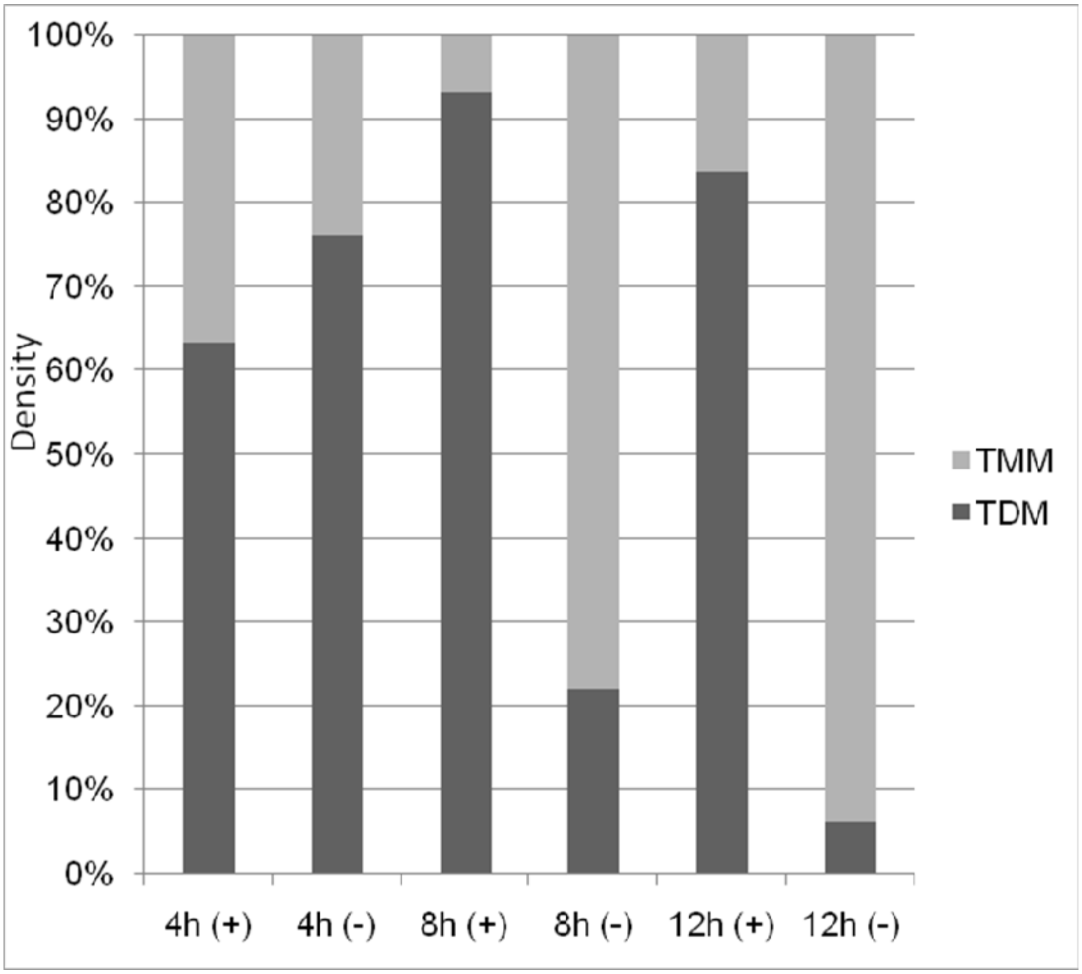

Densitometric estimation of TMM and TDM levels from TLCs shown in Fig.4A, expressed as percentages. (+); cultures grown in the presence of acetamide, (-);cultures grown in the absence of acetamide.

Related to Figure 4A

Figure S2

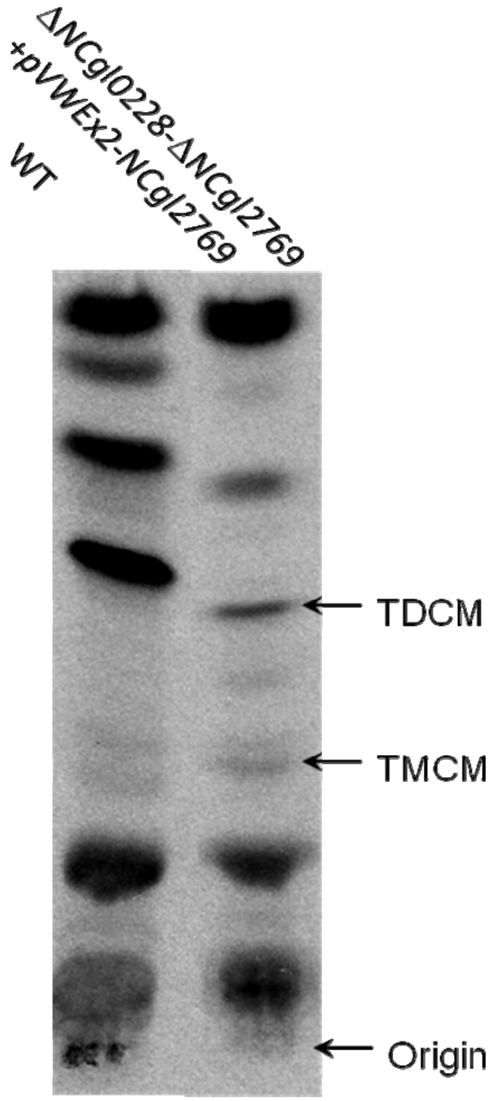

Fractionated total lipid extracts from *C. glutamicum* WT and complemented strain (flow-through fractions from samples applied to a Supleco LC-SAX anion exchange column)

Related to Figure 7A.
